# What works? A qualitative study of participants experiences of a traditional lifestyle intervention with a work focus

**DOI:** 10.1080/17482631.2022.2116988

**Published:** 2022-09-02

**Authors:** Wivi Andersen, Anita Dyb Linge, Chris Jensen

**Affiliations:** a The National Advisory Unit on Occupational Rehabilitation, Rauland, Norway; b Volda University College, Institute of Social Science, Volda, Norway, and Research and Development Manager, Muritunet Rehabilitation Centre, Norway

**Keywords:** Obesity, occupational health, health promotion, weight management

## Abstract

**Purpose:**

Obesity is related to lower labour force participation, increased sickness absence and reduced productivity. The rehabilitation services in Norway have not had much experience introducing a work dimension into lifestyle interventions for persons with obesity. Therefore, this study aimed to evaluate one such type of intervention.

**Methods:**

This is a qualitative study seeking to gather data on the participants’ experiences. Twenty participants were recruited from two lifestyle interventions. Intervention A, with work focus, included lectures and individual guidance from a work consultant in addition to the lifestyle intervention. Intervention B was a traditional lifestyle intervention. Data were collected by semi-structured interviews held at each stay.

**Results:**

Seven main themes emerged and one of them pointed towards a confusion of the aim of the intervention, which was viewed as focusing on lifestyle rather than a process focused on work. Otherwise, the results showed that persons with obesity struggle with many of the same inhibiting factors as other groups with reduced work ability.

**Conclusions:**

The application process might explain the focus on lifestyle change. Communication, guidance and support reduce barriers for lifestyle change, but work is important for general health and social well-being and a work focus may therefore be beneficial in all lifestyle interventions.

## Background

According to the World Health Organization (WHO) the increasing number of people struggling with obesity is a health challenge in most of the world (WHO, 2004; World Health Organization, 2000). The number has trebled for the last thirty years, causing WHO to term this a “global obesity epidemic” (WHO, 2004). The individual costs of obesity are related to reduced quality of life and health problems, both mental and physical health (Han et al., 1998; Kalarchian et al., 2007; Romero-Corral et al., 2006; Wearing et al., 2006). Obesity is also associated with reduced economic and social opportunities, decreased quality of life, as well as comorbidity (Kolotkin & Andersen, 2017). Not only is there a health hazard connected to obesity, it can also affect everyday, practical pursuits and social relations, which again can have consequences for mental health (Avila et al., 2015; Colditz & Dart, 2018). A Norwegian study found that persons with obesity often experience social anxiety and worry about other people commenting or staring at them (Nossum et al.,

Although the prevalence of obesity is lower in Norway than in many other European countries, Norway is also facing a trend of increasing obesity in the population (Meyer & Vollrath, 2017) According to the Norwegian Institute of Public Health , 2017 between 15–21% of children struggle with overweight or obesity, although this trend seems to have levelled out. About 25% young people have overweight or obesity, and in men and women aged between 40–45 years, there are about 25% men and 20% women with obesity. In this group, the proportion with overweight comes in addition to the proportion with obesity (Meyer & Vollrath, 2017).

The level of obesity has shown to be associated with reduced work ability, lower labour force participation, increased sickness absence and reduced productivity (Andersen et al., 2017; Capodaglio et al., 2010; Cawley et al., 2007; Kaleta et al., 2006; Mohammadi et al., 2014; Neovius et al., 2009; Van den Berg et al., 2009). A study from Denmark reported a yearly 1, 8 million extra days of work absence and close to 1.100 cases of disability pension related to obesity (Juel K. & Brønnum-Hansen, 2006). A report from The Organization for Economic Co-operation and Development (OECD) showed that persons with obesity between the ages 50–59 have three times as much work absence as those who do not struggle with obesity (OECD/EU, 2016). This indicates that persons living with obesity have not only health-related challenges but also work-related ones.

While conceptual models of return to work and work disability have been addressed in several journal articles (Kristman et al., 2020; Schultz et al., 2007), these have not focused on obesity and lifestyle change (Bültmann & Siegrist, 2020). However, there have been some studies on the association between work ability and multifactorial lifestyle risk, such as a Norwegian study where low work ability was associated with lifestyle risk factors such as unhealthy diet, low leisure-time physical activity, overweight/obesity and smoking (Oellingrath et al., 2019).

Until now, work focus has not been a part of lifestyle interventions for persons with obesity in Norway, although there is an increasing awareness of the problem of reduced work ability for this group (Linge et al., 2021a, 2021b). In order to meet the challenges that persons living with obesity face regarding their working life, a newly developed work intervention was introduced as an additional element to a lifestyle intervention programme for persons with morbid obesity. This study aimed to identify what the participants experienced as promoting or hindering work participation related to lifestyle, health, work-related factors, and their private situation. In addition, the study sought to identify changes in these areas during the intervention.

## Materials and methods

### Study design

The study design was a prospective qualitative interview study.

### Methodological orientation and theory

The underlying methodological orientation was systematical text condensation (STC; Malterud, 2012, 2017), a method inspired by phenomenology but representing a pragmatic stance. The analysis of the transcribed data was done using an inductive approach based on Malterud’s description of a four-step analytical process (Malterud, 2017): overview and tentative ideas of themes, coding, condensation and recontextualization.

### Participant selection

The participant selection was purposive; the participants were persons diagnosed with morbid obesity and already admitted to the lifestyle intervention by the regional hospital Moere and Romsdal Hospital Trust. As such, the sampling was homogenous (Onwuegbuzie & Collins, 2007).

The admission office of the institution sent out information and request of participation in the study on admission to the lifestyle programme. The participants were then contacted by phone prior to their stay, and asked whether they wanted to participate in the study. Based on oral consent, the participants were randomized to intervention A or B by the Norwegian University of Science and Technology (NTNU). On arrival at the institution (baseline), participants were again informed of the study and asked for written consent.

The aim in the overriding study was to recruit 180 participants, 90 to intervention A and 90 to intervention. In the period 2014–2016, the institution admitted 178 persons; of these 33 did not meet the inclusion criteria; 5 did not wish to participate in the study and the rest (140 persons) gave written consent to participation. A selection of the participants (40 persons) was asked to participate in in-depth interviews. Participants in intervention A (with work focus) was prioritized, and twenty participants were recruited.

The inclusion criteria were a body mass index (BMI) >/ = 40, *or* BMI >/ = 35 with comorbidity such as cardiovascular disease, musculoskeletal conditions, diabetes mellitus type 2, breathing difficulties or hypertension, and age between 18–60 years old. Exclusion criteria were lack of capacity to consent, severe alcohol and/or drug abuse, major mental illnesses, being pregnant, having a health condition that contraindicates physical activity, receiving or having applied for disability benefits, or having disabilities requiring permanently modified work.

### Participant demographics

All twenty participants were interviewed during the intervention (Table 1). Six were lost to follow-up. About half of the participants were working more than 50% of a full-time position at baseline. The remaining were working 50% or less of normal working hours or were on sick leave, receiving work assessment allowance or partial disability pension. A work assessment allowance ensures that you have income during periods when you, due to illness or injury, need help from the Norwegian Labour and Welfare Administration (NAV) to return to work. A majority of the participants had been part of the workforce for more than five years.

**Table 1.** Participant demographics.

Gender	M: 6		F: 14		
Intervention	A: 7		B: 13		
Marital status	Married/cohabitants		Single		
	15		5		
Age(Mean: 40,5)	20–29	30–39	40–49		50–59
	3	8	3		6
Occupation	Service: 11Professional: 5Academic: 2Not working. 2				
Working status before VR	WAA = 100%: 4FWP ≥ 100%: 6, FWP ≥ 50: 2SLB = 100%: 2 *Combined benefits: 5*				
BMI baseline (kg/m^2^)	Mean			Range	
	42,3			30,7–57,2	

FWP = full work participation, SLB = sick leave benefit; WAA = work assessment allowance

### Intervention design

**Figure 1.**
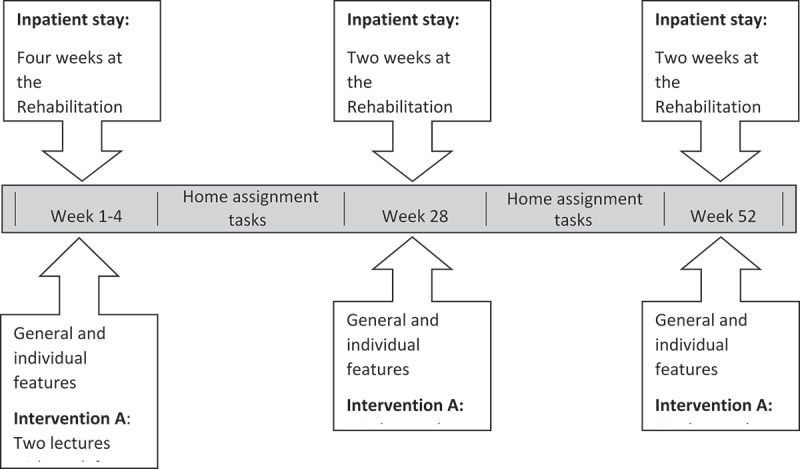
Intervention design.

The lifestyle programme (which equal intervention B) was in-patient and group-based and lasted for a year (Figure 1). Intervention A included, in addition to the lifestyle intervention, a work intervention that aimed to provide the participants with strategies to master challenges to work-life participation, enhancing the individual’s work ability. The participants in intervention A had individual counselling with a work consultant, and at baseline, there were two lectures given by the work consultant. The first, “Duties and rights as employees”, had a focus on possibilities in their work situation as well as the participants’ duties. The second lecture, “Work as medicine”, aimed at raising consciousness on the participants possibilities for returning to work as well as health promoting aspects of work. A career guidance model, cognitive information processing (CIP), was used to mainframe the work dimension. CIP is a triangular model that is designed to systematically explore and talk about knowledge about oneself, about one’s choices, one’s problem-solving strategies and any limiting thoughts that might affect one’s choices with a focus on workability.

All participants received a traditional lifestyle intervention with the aim of lasting lifestyle change through learning health-promoting strategies related to physical activity, diet and cognitive approaches, and skills for coping strategies to obtain behaviour change and increased well-being (intervention B). The multidisciplinary team working with the participants consisted of a nurse, a physiotherapy-specialist, a psychologist, a specialist in occupational therapy, a sports educator, a doctor specialist in pulmonary and internal medicine and a nutritionist.

### Setting of data collection

Qualitative data were collected via semi-structured interviews at three different stages of the intervention: At baseline, after six months and after twelve months. At baseline and the majority of the interviews after twelve months, the participants were interviewed with only the interviewer(s) present, face-to-face in an office at the rehabilitation centre well suited for interviews. The interviews at six months were mainly phone interviews due to practical and logistical reasons. These interviews were set at times that suited the participants, and in the privacy of their own room at the rehabilitation centre. The interviewing researcher conducted the interviews from a suitable office at her own institution sitting alone with locked doors.

### Data collection

Two thirds of the interviews at baseline were carried out by a master student at the Norwegian University of Science and Technology (NTNU) and his supervisor, a senior researcher (PhD) at the Norwegian National Advisory Unit on Occupational Rehabilitation and professor at Department of Public Health and General Practice, Faculty of Medicine, NTNU, Norwegian University of Science and Technology. The rest of the interviews were carried out by a researcher at the Norwegian National Advisory Unit on Occupational Rehabilitation (PhD).

**Box 1.** Topics included in the interview guide

• Background	
• Quality of life	
• Workability	
• Intervention (expectations and experiences)	

A semi-structured interview guide was developed, see Box 1. To explore the participants’ experiences in depth we used descriptive questions as “How have you experienced these last weeks?”

The interviews lasted from 60 to 90 minutes and was recorded with a digital recorder. Each researcher transcribed his or her own interviews apart from seven interviews, which were conducted by a hired transcriber approved by NK-ARR and subjected to a requirement of confidentiality.

Baseline demographic data were collected from all participants before starting the interviews, including age, gender, education, work status and years in working life.

### Data analysis

Data was coded and analysed by one researcher using NVivo, a software program for qualitative research. The first step in Systematical text condensation (STC) entailed to establish an overview of the data and a tentative idea of possible themes. The transcripts were read several times; first, they were read through after transcription, then re-read after all the interviews at each new stage was reached; at completion of all interviews at baseline, at six months and then finally all interviews were re-read at twelve months.

The second step entailed to sort and identify “meaning units” within this material. These “meaning units” are text fragments describing the participants’ experiences of what has hindered or helped them in the process. The “meaning units” were sorted in the categories: Contextual factors hindering; internal factors hindering; contextual factors promoting/helping the process; internal factors hindering the process. After sorting out meaning units, they were coded. For example, were some meaning units representative of participants’ feelings that stress at work hindered them in their process. Through detaching text fragments from the interviews, the second step represents a decontextualization.

The third step entails what Malterud (2017) terms condensation; where the meaning units within the code groups (and subgroups if there is enough data), are rewritten into a first-person narrative, in order to create a synthesis—an overriding meaning—of the meaning units in every code group. Some code groups only entailed very few meaning units, some of these meaning units were instead subgroups of other code groups, while one code group seemed to represent an important finding (abuse) that neither could nor should be integrated within other code groups, and were therefore kept on as a separate code. Experiencing abuse or violation was a finding that was not representative for the group, but never the less important. However, because this finding was atypical and not a general theme, the authors have chosen not to outline it any further.

The fourth step entails a recontextualization. The narratives created in step three formed a starting point for forming descriptions and concepts, that is, they formed the basis for an analytical text relating to every code group. The findings were validated against the “raw” data, which is the original interviews, in order to make sure that the findings represents the experiences told by the participants. This step resulted in themes, and text excerpts that were representative were included as quotations in the report.

The authors have chosen not to contextualize the quotations any further in order to maintain the participants’ anonymity.

Data were analysed on each stage of the process: After completing all interviews at baseline, after six months and after twelve months. As such, there has been an ongoing traverse analysis of data on each stage in order to identify themes. In order to identify changes between the stages, the participants’ process was also followed longitudinally.

### Ethics

This study was approved by the Regional Committee for Medical and Health Research Ethics South-East Norway (REK nr.2014/697/REK South East) and was registered in Clinical Trials with the trial identifier NCT01361490. All written consents to participate in the study followed the laws and guidelines for proper health research and treatment according to the Specialized Health Care Services of Norway. Furthermore, the study was conducted in accordance with the Helsinki Declaration (World Medical Association, 2013) and adheres to the CONSORT guidelines for pragmatic trials.

## Results

Seven main themes emerged from the analyses; one of them was about confusion of the aim of the intervention. The general aim of the lifestyle intervention is to achieve permanent changes in lifestyle by providing strategies for mastery connected to changing habits to reduce comorbidity, improve physical and mental health, and increase participation in social life. Therefore, a requirement for admittance to the intervention is that the patient is motivated for change. The participants leaned towards interpreting their process as a lifestyle process rather than a process towards increased work ability. Furthermore, the other six themes revolved around what hindered and promoted the participants’ lifestyle change process. Three of the themes described experiences hindering their process: 1) injustice; 2) health as a barrier; and 3) to feel different, while three themes represented experiences facilitating the process: 4) recognition; 5) experiencing improved quality of life, and 6) self -confidence through knowledge and coping experiences. Changes during the process (longitudinal changes) were present in all themes, but especially in theme 2, health as a barrier, because the participants experienced significant weight loss and improved quality of life during the year of intervention.

### Confusion of aims: lifestyle change or increased workability?

The participants in the work intervention generally interpreted it as a process of lifestyle change rather than a process to improve their capacity to work. However, the original lifestyle intervention focused on lifestyle change and weight reduction, and this was the overall context for the added work focus. Also, the participants had a significant weight loss during their year of intervention, and for them, this corresponded with improved health, reduced medication, and improved quality of life. The health-promoting results seemed to have redirected the participants’ attention from work to health. For the participants who experienced the work intervention as helpful, work environment issues and work ability were partly focused during the early part of the intervention, especially between baseline and the second in-patient stay. Nevertheless, these participants also shifted their focus at the end of the intervention; from viewing their goal of increased work ability, to a process aimed at weight loss and improved general health.

During the interviews at baseline, the work intervention was not highlighted as especially useful or important. The lectures generally touched upon themes the participants already had knowledge of. However, a few younger employees mentioned that the lectures on work had been important because they were informed of rights and duties in the workplace not formerly known to them.

The counselling with a work consultant was experienced as «ok». The participants got to talk about their work and how they experienced their workday. Though, there were doubts on whether these counselling sessions were going to produce any changes:

*I(interviewer):* *What were you talking with the work consultant about?*

*P (participant):* *Well, about work and such. Whether I enjoyed work or not*

*I:* *Was the counselling helpful to you?*

*P:* *It was not really relevant for me, but it was nice talking to him, anyway.*

*I:* *What was nice with having such a conversation?*

*P:* *It was that I asked him if he could help me stay in my current department at work, because I would be happier there, and closer to home. And I could cycle to work.*

However, for some, this view did change during the intervention. Those having problems at the workplace and who allowed the work consultant contacting their employer to find solutions did experience the contact resulting changes that improved their workday. They felt this change would not have come around without the help of the work consultant because they would not have “had the guts” to address these problems or contact their employer on their own. Support from the work consultant during the intervention gave these participants the confidence to seek changes, and they experienced the counselling sessions as useful:

*I:* *How useful do you think the counselling sessions with the work counsellor has been for you?*

*P:* *I think they have been useful.*

*I:* *In what way?*

*P:* *They helped me get in contact with my employer, and for him to allow me to stay where I want to be.*

Participants experiencing being helped, wished for even more help and counselling with the work consultant in the intervention. However, even though the view on the work intervention and its significance for the individual participant changed, it did not lead to any understanding that the goal of the intervention was to increase work ability. Throughout, the participants view the entire intervention programme as a lifestyle intervention. However, they thought that work focus should be a part of any lifestyle intervention.

### Injustice

At baseline, disrespect was described as a limiting factor. Disrespect entailed experiencing injustice and/or the feeling of not being heard. The participants placed the problem within the family sphere, at the workplace or within the health care system or the labour and welfare system.

The workplace could be experienced as a very inhibiting factor to the process, if the participants felt unfairly treated and/or not recognized. It was especially the relation to the workplace leader which defined whether the workplace was a limiting factor or not. The participants who felt unfairly treated, were demotivated and frustrated. Unjust treatment also seemed to affect how the participants experienced their working environment, because injustice entailed that other employees were treated better.

I like work, but I do not like the place I am working now because of the poor working environment. There is a lot of backbiting and unfair treatment from our leader, so that sucks. After closing hours, we are just two, and in addition to the regular duties, we have to do the odd jobs that there is not time for in daytime, but we can’t do it all. Therefore, you go home feeling guilty, and then the leader yells at you. Then there is a co-worker who always makes mistakes is lazy and cannot be bothered to do anything – she always gets a pat on the shoulder. It is very clear who is the leader’s favorite.

To feel overlooked or ignored, especially by one’s manager, was also a factor that the participants identified as inhibiting. Some felt powerless in their working situation. Powerlessness was also felt when work demands exceeded work capacity. Work demands were experienced as overwhelming due to a variety of reasons (the situation at home, poor health, high-performance pressure at work, or experiencing work as a heavy burden of responsibility). A common denominator was that the participants felt that their situation was not seen or appreciated by their employer or leader resulting in a feeling of inadequacy. A participant working in administration with responsibility for a range of disciplines described it this way:

I am always worried I have overlooked or ignored something, and this is a permanent stress factor for me. I am responsible for the switchboard, the archives and the desk. In addition, I am supposed to supervise and teach the computer system, as well as making routines and plans. It is in a way … it is too much for one person. It really is.

For some, the discontentment with their workplace lasted throughout the whole intervention. The participants in question had a poor relation to their nearest leader, and their organizations were going through a reorganization phase.

For female participants, lack of recognition within the family sphere was also a problem. The women had main responsibility for house and children, and this responsibility came in addition to their work. They felt their partners did not view housework and responsibilities towards children as part of a workload and that their spouses did not take their fair and equal share of these chores. Even if their job did not hinder their process, the chores at home could overthrow the balance between coping or not. One woman stated:

I like my work. It is high paced, but does not stress me out as such, just occasionally. But for some time now, everything that takes place at home has to go through my head first. Other persons schedules, what they are going to do every day. The kids – what they are supposed to wear, and what they are supposed to bring.

Some had children with special needs. This required extra effort regarding procuring medical, pedagogical, or other forms of help from community or government agencies. These women experienced their total workload as a factor that severely hindered their process.

### Health as a barrier

The participants often experienced their health as a barrier to their process. Common ailments were musculoskeletal disorders, diabetes, coeliac disease, high blood pressure, anxiety and depression, worries, and stress. Both working participants, as well as those on sick leave, reported health problems. The ailments represented a hindrance in everyday life, in that the participants had to adjust both work life and home life according to the physical challenges experienced by the participants.

Participants with the most severe health problems and social security agreements from the Norwegian Labour and Welfare Administration (NAV) had major health problems even after a year of intervention. Their main problem throughout the intervention seemed to be the balance between activity and rest, i.e., finding their physical limits:

This year, from the last stay and until now, all I have tried to do is to discover a middle ground. That is, diet and training that works for me. Meaning that I do not over exercise and get enough restitution, so that it works in my everyday life. Because I do have days where I can hardly get out of bed, those are the bad days. Then you have the good days, those where you can be outside all the time. I have had my ups and downs. It has been like that for many years now, so you can say I am used to it.

During the intervention, the stress seemed to fade. Those who still did experience stress lacked improvements in their dialogue with their closest leader. This group also had a prevailing fear of future work ability and work situation due to conflicts or poor dialogue with their nearest leader.

Participants who experienced health as a hindrance often worried about future work and health. These worries were mainly whether the work capacity was sufficient to satisfy demands at work and/or home. These participants were either unemployed or receiving health-related benefits; thus, they were outside the workforce in varying degrees. However, during the intervention, there was a change towards a more positive outlook.

However, obesity and comorbidity did not represent a problem for everyone. A common denominator for participants working full time both before and during the intervention, was that they did not experience health or obesity as a hindrance for work. They had a good dialogue with their employer and had possibilities for organizing their workday according to their health.

### To feel different

One inhibiting factor, especially in physical activity or exercise, was a feeling of being different. It prevented the participants from doing physical training in areas where other people trained, such as gyms or indoor swimming pools. Although most participants experienced the joy of movement in the activities during the first in-patient stay at the institution, they expressed a reluctance for keeping up these activities when returning home:

I used to swim, but I don’t know how many years it is since we got a new swimming pool in town – I haven’t been there. I’ve never been there. I have talked with other people in my group (at the intervention, ed.) and we all feel awkward about going to the indoor pool. There are so many people there, so you don’t feel good about yourself there, do you. Then you rather stay at home. This threshold of feeling a bit different from everyone else make you stay away from such places. I don’t like gyms either. I don’t like to undress with everyone else.

Some made it clear that the shame they felt led to a certain social withdrawal.

### Recognition

An important factor for progress during the rehabilitation process was being listened to and treated with respect. Important factors for enjoying work were a good dialogue with and support from their nearest leader, getting to use their skills and education in their work, being able to handle the workload, and having a good social environment at work. Work was viewed as important for self-esteem contributing in the society.

Some of the women experienced a change in support from their families during the intervention. Their efforts at home were more appreciated, and they received more help at home from their partner and their children. They explained this change with two factors; partly that their workload at home became apparent for their family while away at the institution, and partly that they themselves became aware of their workload at home and demanded greater participation in chores from the family:

*I:* *Last interview you said that you did most of the work in your home. Have this changed since last time?*

*P:* *Yes. I was away for a month last time, so he quickly discovered what I really do at home. …*

*I:* *Can you tell a bit more of what has changed between the stays (between baseline and last stay, authors comment)*

*P:* *More understanding of me being tired, and sharing more on all household chores.*

Participants who experienced support from a social case worker in NAV underlined this support as important. Being met with understanding and a good dialogue was important support during the process. The participants highlighted the importance of their case worker knowing and keeping updated on the content of and their progress in the intervention programme.

Another supportive actor was their general practitioner (GP), an active “co-player” in the process. Lifestyle changes often resulted in weight changes, increased physical capacity and changes in medication. The GP had a necessary and important part to play in the process.

### Experiencing improved quality of life

During the first in-patient stay, the participants experienced improvements in their physical endurance and strength. They saw visible results by losing weight and change in medication. However, the biggest changes were at the second stay, six months into the intervention: They had more energy both at home and at work, and experienced a body in transition—not just losing weight but also gaining muscle mass. They also noted improvements to their other health problems.

When I started the intervention, I had big health problems, so I couldn’t participate in all the activities during the first stay. I was on cholesterol-lowering medication which made my legs ache. I have stopped using them now, because I’m keeping the cholesterol low with diet changes.

Mental health also seemed to improve. Physical improvement gave the participants motivation to stay in the process and feel mastery. Total changes resulted in a more positive view on life for those of the participants who initially had struggled. Summing up the process, a participant stated:

The most important thing with this process is that you experience a positive change in quality of life. You see for yourself it can change – it works.

### Self-confidence through knowledge and coping experiences

The first in-patient stay gave new knowledge of diet, physical activity and mindset. In addition, the participants experienced joy of movement and mastery of new athletic skills through being challenged with new forms of activity or trying out activities they had not done for years.

The concrete results of the gained knowledge were visible at the second and third stay. The participants implemented the knowledge in their everyday lives, making changes in diet and physical activity. Diets were changed by introducing more fish and vegetables; they ate more regularly and planned meals and mealtimes. Further, physical activity was put into system by scheduling training sessions. Even though intensity and frequency would vary a lot between the participants, all increased their everyday activity and training sessions. During the first stay, they gained awareness of the impact of negative thought patterns and learned techniques for stress and pain mastery as well as a positive mindset:

*I:* *I was reminded of it this February, because I have not been doing outdoor walking since November, but when my partner decided that we should go for a walk to a nearby lake, I thought, “This is going to be hard. We might not make it”. Then I thought, “I recognize these thoughts, these negative thoughts. I have to focus on the positive instead”. Despite this, I just got more and more negative, but I walked on despite it and all of a sudden, we were at the lake! Then the positive thoughts came, that it had not been at all strenuous—it was easy as f … So now, I know things ‘aren’t as hard as I think they are. I am still fit even thought I ‘haven’t been walking for three months, but I have been lifting weights. I thought I would be less enduring than I was, but I am in ok form.*

*I:* *If this trip had been before the intervention, do you think you would you have handled it differently?*

*P:* *Yes, because now I knew that the negative thoughts were just thoughts, but I ‘didn’t know that before we had lectures on it on the first stay. Now I know that negative thoughts will often appear, but you just have to ignore them and complete your goal.*

Some underlined the structuring effect work has on everyday life. Despite everyday stress, for them, work provided an underlying structure that made it easier to implement new routines.

## Discussion

### Methodological considerations

One of the aims was to gain more insight into the complexity in the crossing field between morbid obesity and work ability. The participants own voice on what they see as facilitating or inhibiting this process was vital for this insight. The strength of this study has been the choice of a qualitative method to gain insight into these experiences since the aim of qualitative methods is to seek the participants’ experiences of social phenomena. As such, these methods can identify nuances and complexity not otherwise accessible (Malterud, 2001).

Another strength of the study has been the longitudinal aspect. Following the participants through the whole intervention, the changes in their experiences of health, private and work situation, as well as their views and experiences of the intervention, has been accessible.

The relatively large number of participants, 20 persons, has contributed to complexity. The participants differ in work status, family status, educational background, and health status at baseline. These differences provide an insight into what both working participants and participants temporarily outside the workforce experience as promoting or inhibiting their capacity to work and their health.

An ethical and methodological weakness is that half of the participants experienced interviewers change during their intervention. From an ethical point of view, a change of interviewer can be problematic. A qualitative method, such as semi-structured interviews, is based on trust between interviewer and participant. An interviewer change can jeopardize this bond and result in fewer data or data of poorer quality. We also conducted some of the interviews by telephone which was a practical and pragmatic choice. Still, the same methodological and ethical implications will be applicable as mentioned above.

Another weakness of the study is that one single researcher did the analysis. Coding and analyses are thus not scrutinized and viewed from different perspectives or negotiated in plenum. However, it has been pointed out that consensus rarely is a relevant validation criterion in qualitative studies (Malterud, 2017; Morse, 1997). A panel of researchers performing the analysis first provides a diversity of perspectives that gives optimal data use rather than validity (Malterud, 2017).

Malterud describes the chosen method of analysis, STC, as “a pragmatic method for thematically transverse analysis of qualitative data” (Malterud, 2017, p. 97). Data have been analysed on each step of the process at baseline, at six months and after one year, constituting a transverse analysis on each stadium of the process to identify themes before each participant has been analysed longitudinal to identify individual changes. This process satisfies the methodical demands of STC as well as providing a longitudinal perspective.

### Discussion of results

The participants generally interpreted the intervention as a process of lifestyle change rather than improving their capacity to work. The regional hospital trust, Moere and Romsdal Hospital Trust, recruited all participants because they fulfilled the criteria for a lifestyle intervention, and as such, the participants would have had certain expectations. These expectations were focused on weight reduction and health problems and seemed to have overridden the work-related goal of the intervention. Still, lifestyle changes may be an important factor that improves the work ability of persons struggling with obesity due to reduced BMI and comorbidity. As such, any successful lifestyle intervention for persons with obesity might improve their work ability.

In addition, is it a very demanding process to go through a process of lifestyle change. The participants experience the lifestyle change process as so important, that it—at least for some time—takes the focus off work and work ability. However, changes in work participation can also depend on factors not directly affected by health or lifestyle changes, such as relations with managers and co-workers and other workplace factors. Workplace modifications can contribute to increased work ability as well as prevent sick leave (Franche & Krause, 2002; Kristman et al., 2020; Linge et al., 2021a, 2021b; Schultz et al., 2007; Young et al., 2005), and job satisfaction has been shown to be an important factor for well-being at work (Ekberg & Ståhl, 2020; Figueredo et al., 2020; Hees et al., 2012; Linge et al., 2021b; De Vries et al., 2011).

Another question is whether work should be an integral part of any lifestyle intervention. People on or at risk of sick leave from work due to obesity or obesity-related problems may benefit from attending a vocational rehabilitation with a lifestyle intervention regarding to health-related quality of life, reduced BMI as well as increased return to work self-efficacy and reduced work absence (Linge et al., 2021a). Today, Central Norway Regional Health Authority requires a work focus on rehabilitation programmes without specifying details of implementation. Work is argued to be an essential part of a person’s health and social well-being Nøkleby et al., 2015; Van der Noordt et al., 2014; Waddell & Burton, 2006). On the background of this study’s results, we recommend work intervention to be integrated into lifestyle interventions programmes when relevant.

### Limiting factors

The results identified three themes representing the most important limiting experiences for the participants: (1) injustice, (2) health as a barrier, and (3) feeling a bit different.

#### Injustice

The participants’ work-related problems might originate from prejudices or problems with meeting physical demands. There is an association between obesity and musculoskeletal disorders (Colditz & Dart, 2018), and health problems are an important barrier for maintaining paid employment (Van Rijn et al., 2014). Systematic reviews also show that persons struggling with obesity face particular challenges in their work-life, such as being hired less frequently and being promoted to a lesser degree than individuals of average body weight (R. M. Puhl & Heuer, 2009). Nevertheless, these participants did not label their problems as stemming from prejudices or physical challenges. They felt limited when their efforts were overlooked or ignored, when their needs were not taken into consideration or if they felt unfairly treated. The first two themes are common for many persons with danger of or reduced work ability (Van den Berg et al., 2009) and support existing literature where working conditions can contribute to illness, disability and absence (Mather et al., 2015; Rugulies et al., 2007). Unfavourable psychosocial factors are stressors that increase the risk of work-related musculoskeletal problems (IJzelenberg et al., 2004) and the risk of sick leave for persons with musculoskeletal disorders and persons with mental health problems (Janssens et al., 2014; Mather et al., 2015). At the workplace, lack of influence, role conflicts and poor leadership are associated with the risk of long-term sickness absence (Clausen et al., 2012). For most employees, a good work environment is vital for job satisfaction, and the employer is important as a facilitator for that (Lippel, 2020; Loisel & Durand, 2005).

Lack of recognition of tasks and responsibilities within the family sphere was a problem for many women in this study. This finding coincides with the findings of Lund et al. (2005), who found that long-term sickness absence among female employees was associated with role conflict in addition to low reward and poor management quality. Participants who lacked support experienced this as unfavourable for their process. These finds support existing literature for people with non-specific musculoskeletal disorders (Figueredo et al., 2020; Sewdas et al., 2020; De Vries et al., 2011).

We then face the question of whether the limiting factors were related to obesity. People with obesity experience prejudices in society, lack of support within the family, and prejudices and marginalization within the work force and health care system (R. M. Puhl & Heuer, 2009; Malterud & Ulriksen, 2010, 2011; R. Puhl & Brownell, 2001; Rand et al., 2017). The participants did not describe the lack of recognition they felt in the workplace, at home or the welfare or health systems as related to their obesity. Perhaps, the problems some of the participants faced in the workplace had nothing to do with prejudices or that the participants were not aware that what they experienced as lack of recognition was prejudices. Participants could also be reluctant to convey that they were exposed to discrimination.

#### Health as a barrier

The lifestyle intervention addressed issues concerning obesity. Awareness of the programme should address participants’ needs to recognize their experiences of being different. The second theme, health as a barrier, indicate that obesity not necessarily is *the* health problem, but instead focus on comorbid problems such as diabetes, musculoskeletal disorders, cardiovascular diseases and poor mental health. The results (themes 1 and 2) indicate similar problems between people with obesity and those who are exposed to sick leave. In light of the results, the lifestyle intervention with a work focus should address work- and work-related problems instead of obesity and obesity-related problems at the workplace.

#### Feeling different

Feeling different was something the participants saw as a limiting factor for changing their lifestyle. It especially resurfaced in connection to questions about their training between the in-patients stays. The participants experienced problems shopping clothes in regular shops or feeling misplaced in public gyms or indoor pools. Negative thoughts and experiences were attached to their bodies, contributing to stand out or differentiated them from others. Feeling different seemed to be a lasting state of mind for many of the participants. For some, body image seemed to decrease during the process, making them even more dissatisfied with their bodies. Despite weight loss, improvement in body image seemed to dissonance with their expectations. Weight bias might have been internalized, resulting in a persistently negative body image (R. M. Puhl & Heuer, 2009). Weight bias internalization (WBI) is occurring when individuals apply negative weight stereotypes to themselves (Pearl & Puhl, 2018; De Vries et al., 2011; De Wit et al., 2018).

In addition to the feeling of being different, morbid obesity will play a role in how physical activity is experienced. Physical activity may be experienced as unpleasant or painful when a person is unfit. Comorbidity such as musculoskeletal problems may also lead to pain during physical activity. The unpleasantness or pain can make the person ambivalent towards exercise and physical activity, despite knowledge of the gain (Danielsen et al., 2016). These factors represent a challenge for interventions where physical activity and exercise is an essential ingredient. One study of women with obesity and their experiences of physical activity showed that maintenance of an exercise regime is conditioned to find an activity that gives the joy of movement (Synne Groven & Engelsrud, 2010). Training regimes might be easier to commit to due to the participants’ inner motivation rather than performing due to external pressure.

### Promoting factors

Three themes emerged in the process: (1) to feel heard and get support, (2) to experience improved quality of life, and (3) to gain self-confidence through knowledge and mastery

#### To feel heard and get support

Work was experienced as an arena for socialization, where skills and abilities were used and for maintaining self-confidence. Work was seen as a contribution to society, as well as an arena providing recognition of one’s contribution. These findings confirm other findings where work affects the individual’s overall health, psychosocial needs and identity (Dodu, 2005; Franche & Krause, 2002; Kristman et al., 2020; Nøkleby et al., 2015; Schultz et al., 2007; Van der Noordt et al., 2014; Waddell & Burton, 2006; Young et al., 2005).

Some women saw their first stay at the institution as an awareness process, where they became aware of the unfair distribution of chores at home. Awareness during the first stay led them to set boundaries at home, contributing to a more just distribution of chores. In this process, family was redefined from a limiting factor to a promoting factor. Boundary-setting seems to be a part of an empowerment process (Koelen & Lindström, 2005).

Findings indicate that support is an important promoting element for the participants.

With its three stays, the intervention was experienced as a lifestyle changing experience and was for many of the participants viewed as “a new start”. This was the first time they were presented with a comprehensive perspective on lifestyle change, and received support to realize the changes. These findings support existing results where interventions were a place for recognition, an arena for increased self-understanding and a possibility for changing negative lifestyle patterns (Haugli et al., 2011).

#### Gained self-confidence

The concept of empowerment entails developing the skills and self-trust to take charge of one’s own health (Koelen & Lindström, 2005). Through support from health workers, one seeks to initiate a process meant to enhance self-confidence, self-control, knowledge and skills (Koelen & Lindström, 2005). The participants experienced that changed behaviour resulted in improved physical and mental health, contributing to a feeling of mastery. Changed behaviour was noticeable in the family’s level of physical activity, which led to added feeling of mastery. In addition to new experiences, new knowledge led to an increased awareness of own habits and behaviour. Without increased awareness, one can know that something is a wanted behaviour without implementing change. Awareness is therefore vital to reduce the gap between knowledge and action., and is a vital and promoting factor in interventions for persons with obesity (Christiansen et al., 2012).

## Conclusion

Participants in a combined lifestyle and work rehabilitation programme focused on lifestyle change during the intervention. Persons with morbid obesity struggle with similar inhibiting factors such as muscular and skeletal problems, social isolation, and psychiatric diagnoses as other groups with reduced work ability. Experiencing a lack of recognition in different areas of their lives was an important hindrance for the participants, as for many other groups with reduced work ability. Several of the participants who became aware of the problem of injustice and lack of recognition communicated either at work or at home where they were allowed to remove or reduce the restriction. An apparent hallmark of this group was the experience of feeling different, especially in areas where the body was in focus. Extra guidance and support during physical activity in the intervention is important to promote mastery and joy of movement. The participants felt the intervention provided support and increased confidence to pursue physical activity between interventions. The seemingly one-side focus on the lifestyle change might be why improved work ability “faded” as a goal during the intervention, despite the work focus. Nevertheless, work is important for general health and social well-being and a work focus may therefore be beneficial in all lifestyle interventions. A work-focus could be beneficial in existing healthcare interventions, but new interventions could also be designed specifically for patients with a need for a combined work and lifestyle intervention.
